# Efficacy of continuous UV-C_222_ exposure of *Candida auris,* methicillin-resistant *Staphylococcus aureus,* and T1 bacteriophage at two soil levels in hospital patient rooms

**DOI:** 10.1017/ice.2026.10414

**Published:** 2026-05

**Authors:** Richard L. Vincent, Stephanie H. Factor, Laura Rivera Boudla, David Atkinson, James J. McDevitt, Bernard C. Camins

**Affiliations:** 1 Division of General Internal Medicine, Icahn School of Medicine at Mount Sinai, New York, USA; 2 Department of Medicine, Division of Infectious Diseases, Department of Obstetrics, Gynecology, and Reproductive Medicine, Division of Maternal and Child Health, Icahn School of Medicine at Mount Sinai, New York, USA; 3 Department of Medicine, Division of Infectious Diseases, Icahn School of Medicine at Mount Sinai, New York, USA; 4 Icahn School of Medicine at Mount Sinai, New York, USA; 5 MRD Lighting, Queens, Long Island City, USA; 6 Department of Global, Environmental, and Occupational Health, University of Maryland, College Park, USA

## Abstract

**Introduction:**

The spread of *Candida auris* (*C. auris*), methicillin-resistant *Staphylococcus aureus* (MRSA*)* and various viruses in healthcare settings is of global concern. Far-UV-C_222_ reduces the concentration of microorganisms in laboratory settings and can be used directly in patient care rooms at doses safe for human eyes and skin. The effectiveness of UV-C_222_ inactivation of *C. auris,* MRSA and T1 bacteriophage (a viral surrogate) in a hospital setting was studied.

**Methods:**

A partially blinded, cross-over study was conducted of two conditions: intervention, active UV and control, no UV. *C. auris,* MRSA and T1 bacteriophage were inoculated and dried onto stainless steel disc carriers at two soil levels, (0.03% BSA and 5.0% CBS), and placed at 24 locations in two unoccupied, two-bed patient rooms. UV-C_222_ luminaires were placed behind the head of each bed and one in the bathroom for both study rooms. Simultaneous 24-h exposures for both rooms were in random order. Pathogens were processed for cultures.

**Results:**

UV-C_222_ doses exposing the discs ranged from a low of 5 mJ/cm^2^ to high 637 mJ/cm^2^. Under treatment conditions, MRSA showed a 1.0 log reduction in 0.03% soil, *C. auris* showed a 2.6 log reduction in 0.03% soil and a 1.0 log reduction in 5.0% soil and T1 bacteriophage showed a 0.6 log reduction in 0.03% soil.

**Conclusions:**

In patient rooms, continuous UV-C_222_ exposure showed decreased concentrations of *C. auris* (low and high soil), MRSA (low soil), and T1 (low soil). Studies are needed to determine benefits in occupied settings.

## Introduction

Annually, 2.8 million antimicrobial-resistant infections occur in the United States, and more than 35,000 people die as a result.^1^ Environmental surfaces are recognized as points of pathogen transmission.^2^ In healthcare, standard, and enhanced surface disinfection practices are applied to reduce potential pathogen transmission at various soil loads; however, the effectiveness of these practices to reduce hospital-associated infections in patients remains poorly understood.

Even though environmental services personnel complete daily manual disinfection, there can be buildup of soiled surfaces between scheduled disinfection times due to recontamination from pathogen-colonized patients and spread by HCWs.^3^ Studies have been conducted to better understand the impact of standard disinfection plus additional disinfection methods.^4^ Germicidal UV covering the UV-C_200–280_ spectrum has shown to be an important adjunct to manual environmental disinfection, primarily at UV-C_254_ in elimination of pathogens.^5,6^

The use of mobile, whole-room, high-intensity UV-C_254_ sources in unoccupied hospital patient rooms after terminal disinfection has been proposed as a quick and effective way to decontaminate surfaces that may have been thoroughly disinfected.^7–9^ However, direct, high intensity application of UV-C_254_ is not practical throughout the patient’s admission due to exceeding the allowable human safety exposure limits for eye and skin tissue. This issue leads to the question, *“Can built-in far UV-C luminaires placed in an occupied room provide a continuous, safe and effective, far UV-C*_*200–235*_ *dose to significantly decrease microbial load between terminal disinfection leading to better health outcomes?”*

To determine the effectiveness of 24-hour continuous far-UV-C exposure to inactivate microbes on environmental surfaces, we used filtered, Krypton Chloride excimer lamps with a primary wavelength of UV-C_222_. Our test pathogens (*Candida Auris (C. auris)*, methicillin-resistant-*Staphylococcus Aureus* (MRSA), and T1 bacteriophage (T1 Phage) a viral surrogate) are representative of microbes of interest to the hospital infection control community and span a range of susceptibility to inactivation by traditional UV-C_254_.^10–16^ We studied the impact of soiled surfaces, by simulating high and low soil levels.

## Methods and material

**Figure 1.**
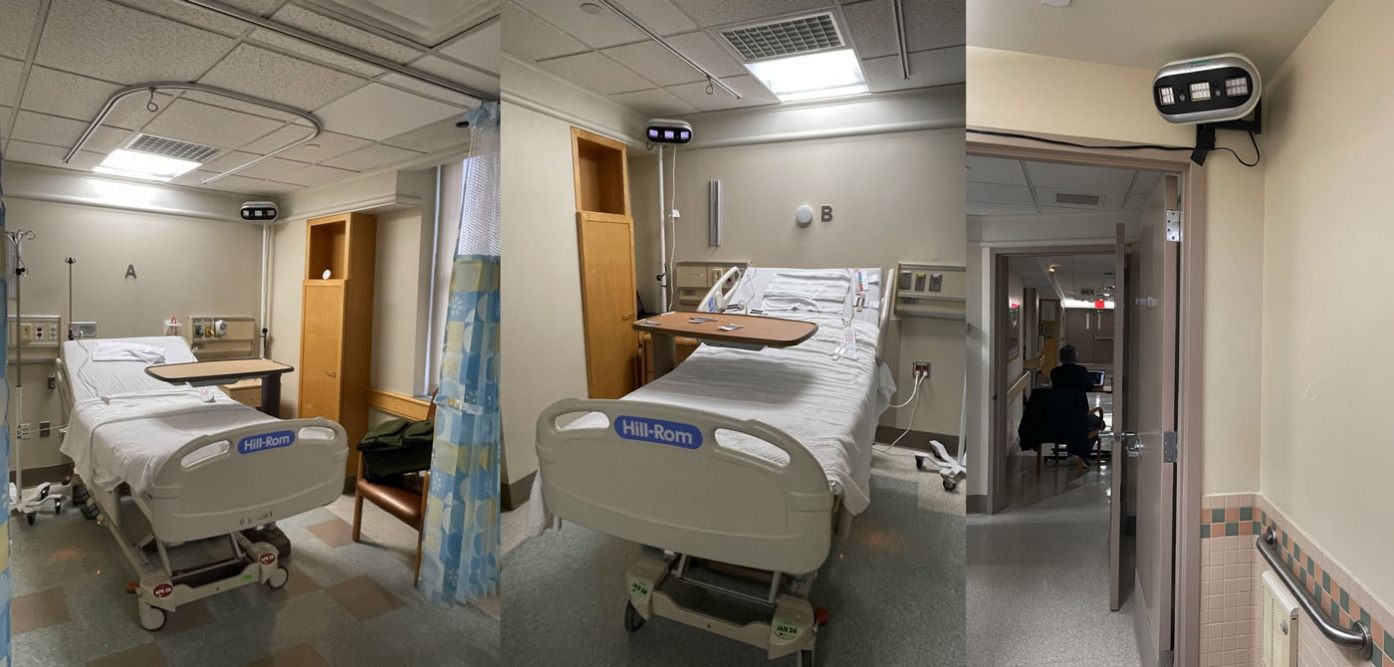
Illustrative room layout with far-UV-C_222_ luminaires placed for the study by each of 2 beds (A and B) in the patient rooms and in each bathroom.

This was a partially blinded, cross over study of two conditions. The intervention condition consisted of exposure from far-UV-C_222_ MTL3 luminaires (Myna Life Technology, Oakland, CA) emitting UV-C_222_ for 24 hours. In the control condition, the specimens were exposed to far-UV-C_222_ MTL3 luminaires that were turned on but emitted no UV for 24 hours. The reference microbiology lab personnel were blinded as to which arm the inoculated discs were assigned. The study was conducted in two double occupancy patient rooms in an unoccupied unit of a hospital in New York City (Figure 1). For the first 24 hours, one room was exposed to far-UV-C_222_, the intervention condition, and the other room received no UV radiation, the control condition. On the second day, the conditions were reversed.

**Figure 2.**
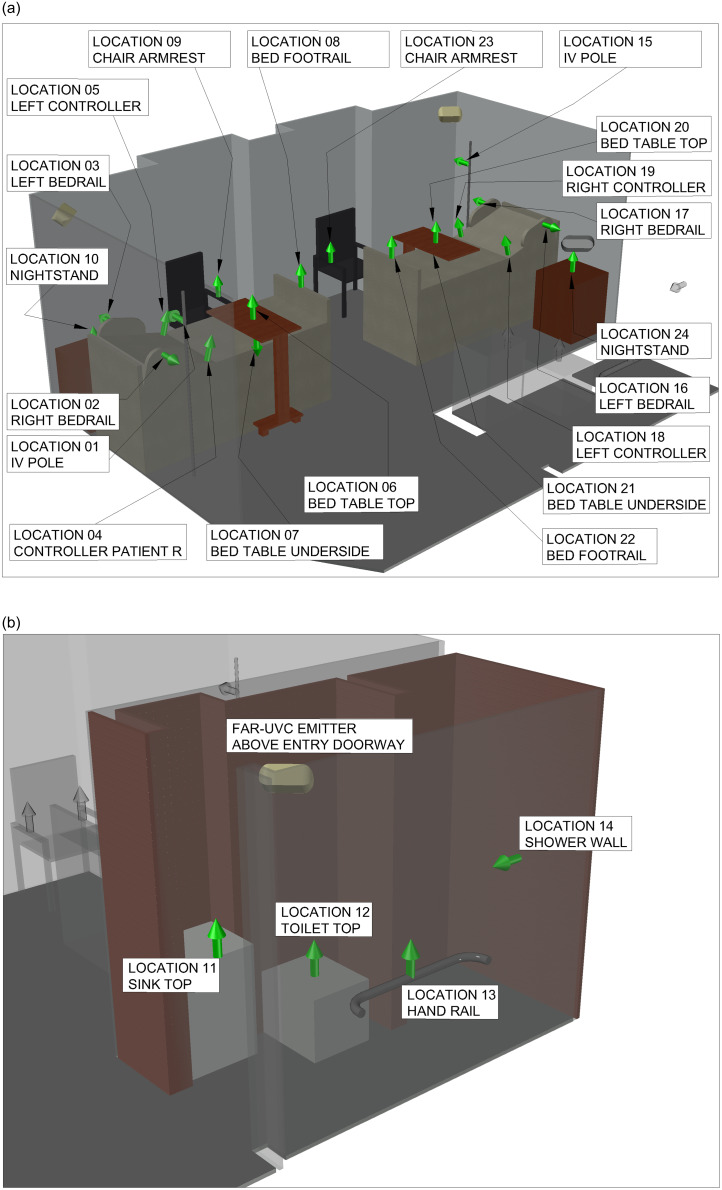
Illustrative 3-D model of placement of 24 inoculated disc carrier clusters in room 2a and in the bathroom 2b. MLT3 UV-C 222 fixtures behind patient the other is shown in the bathroom. All sites covered were recommended by hospital environmental staff. Arrows indicate the vertical or horizontal orientation of the disc carrier clusters mounted with magnetic tape on stiff cards.

Lighting software (Visual, Acuity Brands Lighting, Conyers, GA) was used to model the available luminaires’ placement in each room (Figure S-1), one luminaire was placed behind the head of each bed (total 4 luminaires) and one luminaire was place in each bathroom above the doorframe (total 2 luminaires) (Figure 1). These locations were chosen to maximize the amount of UV distributed in the room and minimize the amount of UV hitting a potential patient’s eyes. Based on previous interviews with hospital environmental services staff, 24 high touch surfaces (see Figure 2a-b) in each room were identified. All sites were directly exposed except four sites shadowed or blocked by bed position (two bed side rail control panels and two undersides of overbed tables).

The 6 luminaires were turned on to determine the amount of UV-C_222_ irradiance received at each of the 24 sites in each room. Handheld calibrated radiometers, X1_1_Optometer, UV 3737-5 Detector set at 222 nm, (Gigahertz-Optik, Türkenfeld, Germany) and Ushio Care_222_ UIT2400, (International Light Technologies, Peabody, MA) measured the amount of UV-C_222_ irradiance and the 24-hour dose was calculated.

The disc carriers (Figure S-2) inoculated with pathogens for the study were prepared by a reference lab (ResInnova, Rockville, MD) using a modified American Society for Testing and Materials (ASTM) standard quantitative disk carrier test method (ASTM E2197).^17^ The test organisms used included: *Candida auris* (CDC#B11903), methicillin-resistant *Staphylococcus aureus* (MRSA) (ATCC#33591) MRSA and T1 bacteriophage (T1 Phage) (ATCC#13303B1) at low and high soil levels. The low soil level represented a contaminated surface after manual disinfection and the high soil level represented a contaminated surface between manual disinfection. For the low soil level, the organisms were suspended in modified soil of 0.03% bovine serum albumin, and for the high soil level, the organisms were suspended in solution of PBS with modified soil of 5% calf bovine serum (CBS).^18,24^ After suspending the test organisms in solution, 0.010 mL was placed and spread to within 1 mm of the edges, and dried on 20 mm stainless steel disc carriers. The reference lab prepared magnetic cases which contained one variety of test organism for each of the 6 conditions. The magnetic cases were placed on ice packs and shipped to the New York City hospital in insulated boxes. Prior to the study, the stability of specimens in low soil inoculum was conducted (see Supplemental File, S-1).

For the cross over study, prior to the study, the disc carriers inoculated with organisms were brought to room temperature over a period of 1 hour. After reaching room temperature, for the intervention condition, the disc carriers inoculated with organisms were transferred to stiff cards (Figure S-2). Each stiff card had 6 labeled circles in which magnetic tape was placed for adhesion. Each inoculated disc carrier was transferred from the magnetic cases surface to the stiff cards using decontaminated, rubber tipped AFM disc grippers (Electron Microscopy Sciences, Hatfield, PA) by study personnel wearing masks, gloves, and gown to prevent contamination (Figures S-2). For the control condition, the organism inoculated disc carriers were kept in open magnetic cases.

On day 1 of the study, 24 stiff cards with the 6 inoculated disc carriers were placed in the intervention room at the previously identified sites and 24 magnetic cases with the 6 inoculated disc carriers were placed in the control room at the previously identified sites. The luminaires in the intervention room emitted UV-C_222_ for 24 hours and the luminaires in the control room emitted no UV-C_222_ for 24 hours. In both rooms, the overhead lights in the patient room and the bathroom were turned on. The blinds in both rooms were drawn to exclude sunlight. Privacy curtains were pulled around each patient bed. The doors to the rooms were closed and locked for the full 24-hour period. On day 2 of the study, the original intervention room was designated the control condition and vice versa.

Rooms with the intervention condition were monitored for the two 24-hour periods to assure appropriate functioning of the luminaires. The control condition luminaires were measured to ensure no UV-C_222_ was being emitted from the sham UV lamps. Three previously described handheld radiometers with UV-C_222_ rated sensors were placed in the intervention room; one on each patient table (n = 2) and one on the toilet seat in the bathroom (n = 1). The three UV-C radiometer results were recorded for 24 hours.

Both rooms had additional environmental monitoring during the study. Supply air and exhaust airflow was measured in both patient rooms and bathrooms using Shortridge AMD-870 Air Data Multimeter attached to Shortridge 8,400 Series flow hood with a 2’x2’ aperture (Shortridge Instruments, Scottsdale, AZ). CO_2_, temperature, and relative humidity were monitored by a Wave Mini air quality monitor (Airthings, Oslo, Norway).

At the end of each 24-hour period, the stiff cards were collected from the intervention room and the magnetized cases were collected from the control room. Inoculated disc carriers on the stiff cards were returned to their original individually number magnetized cases in the same order in which they had been removed. The inoculated discs were shipped overnight to the reference lab in insulated boxes for analysis. The reference lab was blinded to condition of exposure of the individual cases. For lab processing, the organism on each disc carrier was recovered, serially diluted, and plated using standard microbiology techniques to provide quantifiable viable microbial burden.

### Statistical analysis

The paired Wilcoxon signed-rank test was used to compare treated disc carriers to their corresponding control disc carriers for each of the three organisms (MRSA, *C. auris*, and T1 Phage) under each of the two soil levels (0.03% and 5%) because the Wilcoxon signed-rank test is best for comparing paired data which is non-normally distributed. For those comparisons in which the treated condition was significantly different from the control condition, linear regression (with both ordinary least squares and with bootstrapping with 5,000 samples) with log Colony Forming Units (CFU) of the organism as the outcome and UV-C_222_ dose as the exposure was done to provide a quantitative estimate of the effect of UV dose on organism kill.

A *P*-value of ≤ .05 was considered statistically significant. SAS software version 9.4 (SAS Institute: Cary, NC, USA, 2016) was used for all data analysis.

## Results

Exposure to far-UV-C_222_ was significantly associated with a decreased number of CFUs of MRSA in 0.03% soil, *C. auris* in 0.03% soil, *C. auris* in 5% soil, and T1 Phage in 0.03% soil (Table 1).

**Table 1.** Comparison of Colony Forming Units (CFU) on treated disc carriers versus CFU on control disc carriers using Wilcoxon ranked-sum test for paired non-normally distributed data

Organism and soil	Treated median CFU (IQR)	Control median CFU (IQR)	*P*-value^a^
MRSA 0.03%	0 (0, 25)	50 (0, 1150)	.004
MRSA 5%	600 (150, 4850)	350 (0, 43,500)	.19
Candida auris 0.03%	1375 (300, 7100)	28,500 (6125, 123,500)	<.001
Candida auris 5%	6675 (1850, 19,750)	17,500 (8700, 127,000)	<.001
T1 Bacteriophage 0.03%	550 (250, 1525)	6400 (3200, 68,000)	<.001
T1 Bacteriophage 5%	14,500 (4150, 30,250)	3125 (1175, 240,000)	.49

^a^Results of Wilcoxon ranked-sum test for paired data non-normally distributed data.

**Figure 3.**
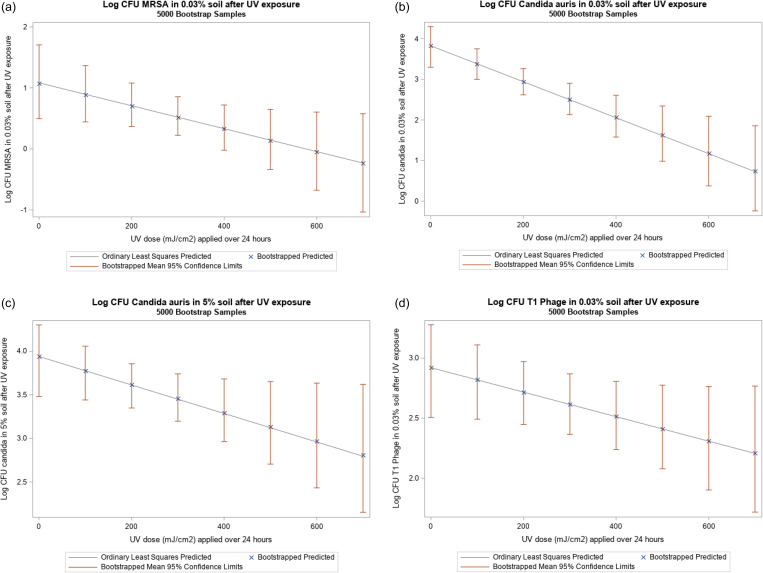
Linear regression (with ordinary least with squares and 5,000 bootstrapped samples) with UV dose as the exposure and log CFU of: a) MRSA in 0.03% soil as the outcome. b) *Candida auris* in 0.03% soil as the outcome. c) *Candida auris* in 5% soil as the outcome. d) T1 Phage in 5% soil as the outcome.

Because the UV doses achieved for this study ranged from 5 to 637 mJ/cm,^2^ the log reductions noted below are for the range between 0 and 600 mJ/cm^2^. The linear bootstrapped regression model with log CFU of MRSA in 0.03% soil as the outcome and UV dose as the exposure showed that a dose of 600 mJ/cm^2^ UV-C_222_ is associated with a 1-log reduction in CFU of MRSA in 0.03% soil (Figure 3a). The linear bootstrapped regression model with log CFU of *C. auris* in 0.03% soil as the outcome and UV dose as the exposure showed that a dose of 600 mJ/cm^2^ UV-C_222_ is associated with a 2.6-log reduction in CFU of *C. auris* in 0.03% soil (Figure 3b). The linear bootstrapped regression model with log CFU of *C. auris* in 5% soil as the outcome and UV dose as the exposure showed that a dose of 600 mJ/cm^2^ UV-C_222_ resulted in a 1-log reduction in CFU of *C. auris* in 5% soil (Figure 3c). The linear bootstrapped regression model with log CFU of T1 Phage in 0.03% soil as the outcome and UV dose as the exposure showed that a dose of 600 mJ/cm^2^ UV-C_222_ is associated with a 0.6-log reduction in CFU of T1 Phage in 0.03% soil (Figure 3d). Again, the regression lines provide estimates; the true value of the reduction lies within the 95% CI (Figures 3a,b,c,d, Table 2).

**Table 2.** Results from ordinary least squares and 5000 bootstrapped sample linear regression with log CFU of organisms as the outcome and UV-C_222_ dose (mJ/cm^2^)

UV dose over 24 hours	Pred value (OLS)	Estimate lower bound 95% CL (OLS)	Estimate upper bound 95% CL	Mean estimate (Boot)	Estimate lower bound 95% CL (Boot)	Estimate upper bound 95% CL (Boot)
**Log CFU MRSA in 0.03% soil after UV exposure**						
0	1.08	0.53	1.63	1.08	0.50	1.71
100	0.89	0.47	1.32	0.89	0.44	1.37
200	0.71	0.34	1.07	0.70	0.37	1.08
300	0.52	0.10	0.94	0.52	0.22	0.85
400	0.33	−0.22	0.88	0.33	−0.02	0.72
500	0.14	−0.57	0.86	0.14	−0.33	0.65
600	−0.04	−0.94	0.85	−0.05	−0.68	0.60
700	−0.23	−1.31	0.85	−0.23	−1.03	0.58
**Log CFU *Candida auris* in 0.03% soil after UV exposure**						
0	3.83	3.32	4.33	3.83	3.30	4.30
100	3.39	3.00	3.77	3.39	3.00	3.75
200	2.94	2.61	3.28	2.95	2.62	3.27
300	2.50	2.12	2.88	2.51	2.13	2.90
400	2.06	1.55	2.56	2.07	1.58	2.61
500	1.61	0.96	2.27	1.63	0.99	2.35
600	1.17	0.35	1.99	1.19	0.38	2.09
700	0.73	−0.26	1.72	0.75	−0.24	1.86
**Log CFU *Candida auris* in 5% soil after UV exposure**						
0	3.94	3.54	4.34	3.94	3.48	4.30
100	3.78	3.47	4.08	3.78	3.44	4.06
200	3.61	3.35	3.88	3.62	3.35	3.86
300	3.45	3.15	3.75	3.46	3.20	3.74
400	3.29	2.89	3.68	3.29	2.96	3.68
500	3.12	2.61	3.64	3.13	2.71	3.65
600	2.96	2.31	3.60	2.97	2.43	3.63
700	2.80	2.01	3.58	2.81	2.15	3.62
**Log CFU T1 phage in 0.03% soil after UV exposure**						
0	2.92	2.51	3.33	2.92	2.51	3.28
100	2.82	2.51	3.13	2.82	2.49	3.11
200	2.72	2.45	2.99	2.72	2.45	2.97
300	2.61	2.31	2.92	2.62	2.36	2.87
400	2.51	2.11	2.92	2.51	2.24	2.81
500	2.41	1.89	2.94	2.41	2.08	2.78
600	2.31	1.65	2.97	2.31	1.90	2.76
700	2.21	1.41	3.01	2.21	1.72	2.77

OLS: based on ordinary least squares analysis; CL: confidence limits; BOOT: based on 5000 bootstrapped samples.

The calculated amount of UV-C_222_ dose (measured irradiance in µW/cm^2^ X 86,400 seconds/day) for each of the 24 sites in each room is presented in Supplementary Table 2. Monitoring of the UV-C emitted during the study did not show any lapses in output during the 24-h exposures as recorded internally by the radiometer or by direct link to a computer. Variation in UV-C_222_ dose at different locations was attributed to distance from the MLT3 luminaire and direct line of sight exposure of inoculated disc carriers vs. blocked or reflected. For example, some inoculated disc carriers were fixed under the patient overbed table which received minimal dose.

Supplemental Table 2 summarizes UV continuous measurements during each experiment.

Air exchange rates for the rooms varied from 4 ACH to 3 ACH.

During the experiments normal winter prevalent conditions were 71–72 °F and low RH from 44–45%.

## Discussion

Far-UV emitting technology in the UV-C_200–235_ spectrum is antimicrobial, inactivating pathogens in air and liquid suspensions.^19,20^ Further, far-UV-C_200–235_ is shorter in wavelength than UV-C_254_ limiting penetration of human tissue, both skin and eyes.^21^ Recently the American Council of Governmental Industrial Hygienists (ACGIH) updated their guidance which previously allowed a Threshold Limit Value (TLV) of 23 mJ/cm^2^ of UV-C at 222 nm (for both eyes and skin) to 160 mJ/cm^2^ for eyes and 479 mJ/cm^2^ for skin over 8 hours.^22^ This ACGIH TLV change allows for higher irradiance levels and associated inactivation of pathogens, with lower health effects compared to the more commonly used UV-C_254_. However, the lack of data on effectiveness of far-UV-C technology in real-world settings has limited its use.

The non-penetrating nature of Far-UV-C_200–235_ may limit its ability to penetrate and reduce microbial loads for surfaces soiled with biofilms. Environmental biofilms either hydrated or dry can act as protective nutritional reservoirs allowing various pathogens, depending on the intrinsic nature of the microbes, to survive environmental stresses over time.^23^ Currently no germicidal UV antimicrobial efficacy standard exists. As a benchmark the EPA standard for chemical agents commonly uses a 5% organic soil level, where a reduction of 99.9% (>3 log) could be considered clinically relevant.^24^

Our findings apply primarily to the selected high-touch surfaces, with largely unobstructed surfaces, in two unoccupied rooms which show that exposure to UV-C_222_ decreased survival of MRSA, *C. auris,* and T1 Phage at 0.03% soil (condition consistent with manual disinfection) and *C. auris* at 5% soil (condition consistent with no disinfection). The study was conducted in real hospital rooms as opposed to a controlled environment in a laboratory. Compared to organism without exposure to UV-C_222_, 600 mJ/cm^2^ of UV-C_222_ was associated with a 1-log CFU decrease of MRSA in 0.03% soil, a 2.6-log decrease of *C. auris* in 0.03% soil, and a 1-log decrease of *C. auris* in 5% soil. Compared with no exposure to UV-C_222_, 600 mJ of UV-C_222_ was associated with a 0.6-log reduction of T1 Phage.

To date, there have not been any other studies that have evaluated the impact of UV-C_222_ on organisms at 0.03% soil in a real-world setting. Future research may confirm our findings.

Memic *et al.* evaluated the impact of UV-C_222_ on MRSA and *C. auris* at 5% soil.^25–27^ In evaluating the impact of UV-C_222_ on MRSA, Memic *et al.* used a real patient room with two MTL3 luminaires in different locations compared to our study and in a much smaller room (35 m^3^ vs. 65 m^3^). They found that UV-C_222_ when applied with the increased intensity was associated with a decrease in CFU of MRSA.^25^ We did not find a decrease in CFU of MRSA at this soil level.

In evaluating the impact of UV-C_222_ on *C. auris,* Memic *et al.* used a laboratory with 2 MTL3 luminaires and in a much smaller room than our study (25 m^3^ vs. 65 m^3^).^24–25^ Similar to our study, they found that UV-C_222_ was associated with decrease in CFU of *C. auris.* Together these studies suggest that UV-C_222_ reliably inactivates *C. auris* at soil levels consistent with the absence of manual disinfection.

In our study, the impact of UV-C_222_ was different at different levels of soil. Ratliff *et al.* conducted a series of laboratory experiments to test the impact of inoculum composition on the germicidal effectiveness of UV-C_254_ and UV-C_222._ They found the presence of 222 nm UV absorbing substances (i.e., salts, sugars, proteins) and dried versus wet inoculum on surfaces can reduce the germicidal efficiency of UV-C.^28^ Those laboratory experiments are consistent with our real-world study. Dried inoculum concentrates both the organisms and soil agent, reducing the penetration of the shortwave UV-C_222_. This may indicate the potential of UV-C_222,_ as an adjunct to manual disinfection, to impact various organisms within a dried biofilm in an occupied patient room.

The impact of the decrease of organisms due to UV-C_222_ on health is not known. Previous researchers have suggested that a ≥3-log reduction in organisms is clinically meaningful; however, studies to demonstrate this have not been done and are needed.^29^ The clinical relevance of our results cannot be determined. However, our results do show that within the limitations of room furnishings and luminaire configuration of our real-world environment, UV-C_222_ is associated with a decrease in organism load.

We found that *C. auris* was more resistant to UV-C_222_ inactivation than was MRSA or T1 Phage. The greater intrinsic resistance of the *C. auris* organism may have allowed the impact of UV-C_222_ to register at both soil levels over a long period of time, whereas, both MSRA and TI Phage showed less ability to survive environmental stresses and register the full impact of UV-C_222._

Our study limitations included: availability of number of MLT3 luminaires to try different, more uniform distributions of UV in the unoccupied spaces, the number of surfaces to be include for these clustered inoculum-based pathogen experiments, and the degree to which microbial inoculation on carriers represent ambient conditions in occupied patient rooms. A future inoculum-based refinement would allow the control carriers to be mounted on individual stiff cards eliminating any potential modification of the microenvironment by magnetic carry cases. For this unoccupied microbial study, potential eye and skin level exposure doses were not recorded; however, in rooms to be occupied, safety assessments should be taken at eye height while seated (in chairs, beds or toilets), laying down and standing, and adjusted (if needed) before occupancy.

This study shows that as an adjunct to standard disinfection, continuous UV-C_222_ surface exposure may be effective for improving hygienic conditions to decrease hospital-acquired infections for patients. Further longitudinal tests with improved UV distribution and ambient organisms in occupied patient rooms with complexities of added medical equipment and extended surfaces are needed to determine the baseline levels of microbial surface contamination and the benefit of continuous UV disinfection between manual disinfection.

## Supporting information

Vincent et al. supplementary material Vincent et al. supplementary material

## Supplementary material

The supplementary material for this article can be found at https://doi.org/10.1017/ice.2026.10414.
