# Supplementary material for: Efficacy of continuous UV-C222 exposure of Candida auris, methicillin-resistant Staphylococcus aureus, and T1 bacteriophage at two soil levels in hospital patient rooms
Source: Infect Control Hosp Epidemiol. 2026 Mar 26;47(5):509–16. doi: 10.1017/ice.2026.10414 (PMC13216793; doi:10.1017/ice.2026.10414)
Supplement: Vincent et al. supplementary material [file S0899823X26104140sup001.docx]

S-1 Supplement File, Figures and Tables

**Stability of Specimens in Low Soil Inoculum**

Before the full study, the stability of the low soil condition was tested. A sample pack consisting of 5 samples of all 3 organisms at 0.03% soil BSA was prepared and shipped from the reference lab to the New York City hospital. The sample pack was removed from the container to allow acclimation to room temperature over a 1-hour period, then the sample pack was opened and exposed to ambient control conditions with blinds closed, room lights on and no UV in one of the study rooms for 24 hour. The next day the sample pack was closed, placed on ice packs, and shipped overnight to the reference lab. The lab tested the 15 samples (5 samples of each of the 3 organisms) and confirmed their patency.

At the lab, each of the 5 carriers per organism at 0.03% BSA were cultured. MRSA showed the greatest stability with initial inoculum concentration of log (CFU/disc) = 10.03 log with the 5 carriers ranging from (7.17, 7.53) a loss of 2.86 to 2.5 logs. *C. auris* initial inoculum concentration log (CFU/disc) = 8.69 log with 5 carriers ranging (5.17, 5.53) for losses of 1.52 to 3.24 logs. T1 bacteriophage at 0.03% inoculum log (CFU/disc) = 7.98 log with 5 carriers ranging (4.18 to 4.48 with on below the limit of detection) for losses of 3.80 to 3.50 logs. The lab reported the exception for the failed T1 outlier, the range between carriers in each strain set was only ~0.36 log. For the full study the lab used these results to adjust the concentrations of the inoculum to have enough range to show an effect of the intervention.

Figures S 1-2


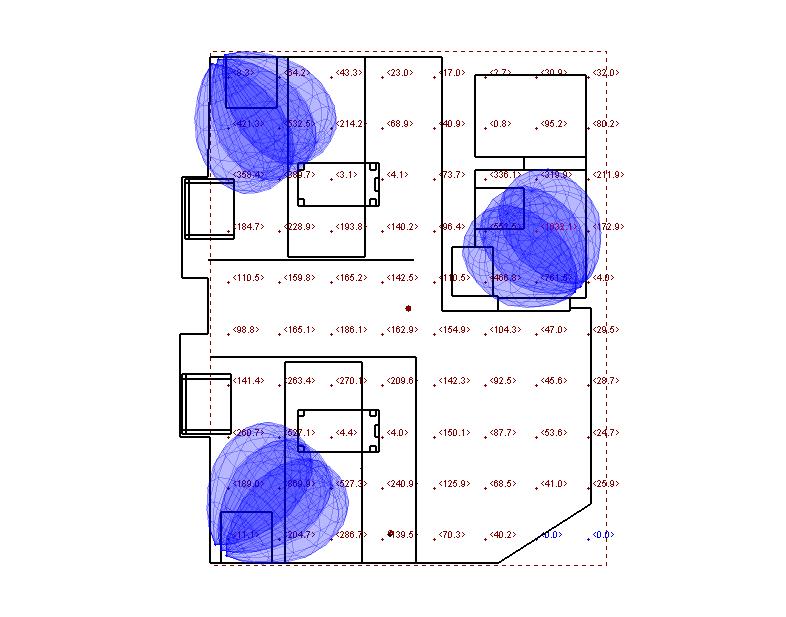


**Figure S-1 Example of UV-C luminaire placement and distribution using lighting software (Visual, Acuity Brands Lighting, Conyers, GA).**


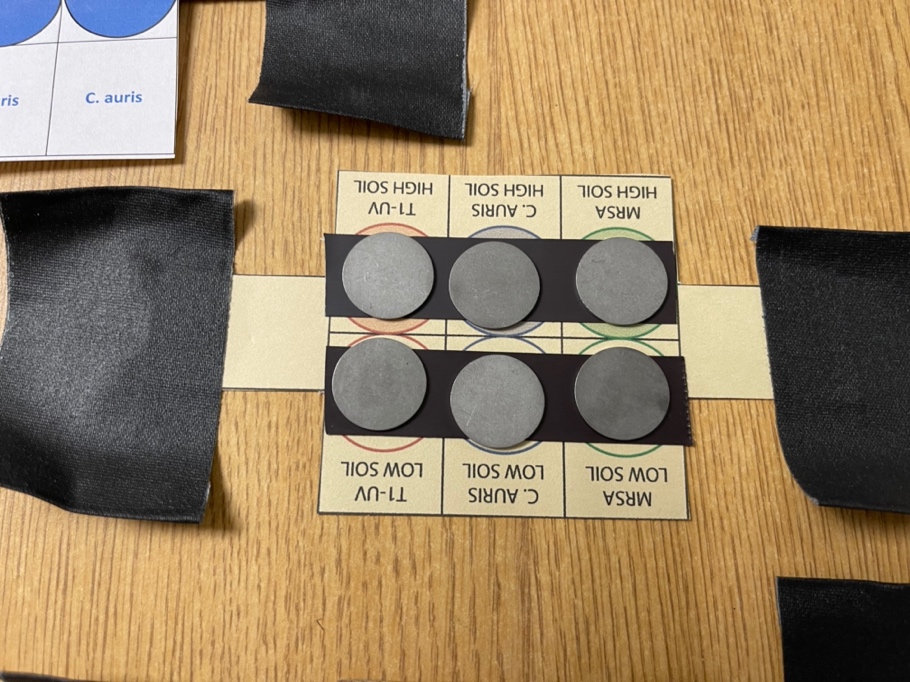


Figure S-2. Image of stiff card with 6 stainless steel discs (SSD) carriers in the labeled circles. Discs were attached to the card using metalic tape.

| Table 1. Dose of UV-C_222_ by site by room over 24 hours (calculated by measuring the irradiance and multiplying by 86,400 seconds per day) | | | | |
| --- | --- | --- | --- | --- |
| **Organisms**  **Location #** | Area | Description | Calculated 24-hour dose of UV-C_222_ | Calculated 24-hour dose of UV-C_222_ |
|  |  |  | Room 9D-62 A | Room 9D-60 A |
|  |  |  | UVC mJ/cm2 | UVC mJ/cm2 |
| **1** | Bed B | IV Pole | 451 | 495 |
| **2** | Bed B | Bedside Rail outside Patient R | 254 | 199 |
| **3** | Bed B | Bedside Rail outside Patient L | 7 | 7 |
| **4** | Bed B | Controller Patient R | 346 | 461 |
| **5** | Bed B | Controller Patient L | 125 | 101 |
| **6** | Bed B | Bed Tabletop | 226 | 239 |
| **7** | Bed B | Bed Table Bottom | 15 | 18 |
| **8** | Bed B | Bed Foot Rail | 98 | 98 |
| **9** | Bed B | Chair arm rest | 64 | 70 |
| **10** | Bed B | Nightstand Top | 23 | 22 |
| **11** | Bath | Sink Top | 523 | 588 |
| **12** | Bath | Toilet Top | 460 | 513 |
| **13** | Bath | Handrail opposite toilet | 567 | 637 |
| **14** | Bath | Shower wall | 169 | 184 |
| **15** | Bed A | IV Pole | 18 | 9 |
| **16** | Bed A | Bedside Rail outside Patient L | 310 | 278 |
| **17** | Bed A | Bedside Rail outside Patient R | 5 | 5 |
| **18** | Bed A | Controller Patient L | 135 | 124 |
| **19** | Bed A | Controller Patient R | 186 | 216 |
| **20** | Bed A | Bed Tabletop | 292 | 305 |
| **21** | Bed A | Bed Table Bottom | 23 | 28 |
| **22** | Bed A | Bed Foot Rail | 98 | 107 |
| **23** | Bed A | Chair Arm Rest | 133 | 149 |
| **24** | Bed A | Nightstand Top | 132 | 138 |

Table 2 Comparison between Estimated dose and Measured Dose (mJ/cm^2^) during all experiments*.*

| **Experiment # ->** | **1** |  |  | **1.5** |  |  | **2** |  |  |
| --- | --- | --- | --- | --- | --- | --- | --- | --- | --- |
| **Location** | GHz est. dose | Avg. est. dose | 24h meas. dose | GHz est. dose | Avg. est. dose | 24h meas. dose | GHz est. dose | Avg. est. dose | 24h meas. dose |
| **Bed A Tabletop** | 308 | 344 | 275 | 308 | 344 | 290 | 291 | 292 | 251 |
| **Bed B Tabletop** | 280 | 347 | 196 | 280 | 347 | 203 | 221 | 226 | 182 |
| **Bathroom wall/shower** | 184 | 204 | 288 | 184 | 204 | 296 | 165 | 169 | 273 |

UV Measurements continuously taken during the course of the experiments with 1 radiometer place in the bathroom, and 1 on each patient tabletops (n=2) to capture cumulative UV at three witness points.

These values show a difference between calculated dose based on irradiance measurements x time in seconds over 24 h vs cumulative dose from direct measurements over 24 h times. The measurements show a lower output of UV-C on Bed B Tabletop than Bed A. The bathroom doses were higher than the estimated dose because the sensor was mounted on a tripod and aimed at the MTL3 luminaire vs direct dose on the actual wall. The witness point measurements were a measure to ensure that accumulated dose could be recorded in case of a power disruption.
